# Hot Yoga: A Systematic Review of the Physiological, Functional and Psychological Responses and Adaptations

**DOI:** 10.1186/s40798-025-00917-7

**Published:** 2025-10-01

**Authors:** Ashley G. B. Willmott, Carl A. James, Matthew Jewiss, Oliver R. Gibson, Franck Brocherie, Jessica A. Mee

**Affiliations:** 1 The Cambridge Centre for Sport and Exercise Sciences (CCSES), Anglia Ruskin University (ARU), Cambridge, UK; 2 Department of Sports and Health Sciences, Academy of Wellness and Human Development, Faculty of Arts and Social Sciences, Hong Kong Baptist University, Kowloon Tong, Hong Kong China; 3 Centre for Physical Activity in Health and Disease (CPAHD), Division of Sport, Health and Exercise Sciences, Brunel University, London, Uxbridge, UK; 4 Laboratory Sport, Expertise and Performance (EA 7370), French Institute of Sport (INSEP), Paris, France; 5 School of Sport and Exercise Science, University of Worcester, Worcester, UK

**Keywords:** Hot yoga, Yoga, Bikram, Exercise, Heat stress, Health, Physical activity

## Abstract

**Background:**

Hot yoga is a collective term used to classify any form of yoga undertaken in warm to hot ambient conditions (≥ 25 °C). This study systematically reviewed the literature concerning hot yoga, with particular focus on acute responses to a single session and identifying prospective health benefits associated with physiological, functional and psychology adaptations following chronic practice.

**Methods:**

The review was conducted in accordance with the Preferred Reporting Items for Systematic reviews and Meta-Analyses (PRISMA), with searches performed across two main databases (PubMed and SCOPUS). Studies were included if they met the Population, Intervention, Comparison, and Outcome (PICO) criteria, were of English language, peer-reviewed, full-text original articles, and using human participants.

**Results:**

Forty-three studies investigated the effects of hot yoga (both acute and/or chronic), totalling 942 participants (76% female). The most common method of yoga performed in hot conditions was Bikram (74%), followed by generalised hot yoga (19%), Hatha (5%) and then Vinyasa (2%). Typical session duration ranged 20–90 min and occurred within 30–52 °C and 20–60% relative humidity. Hot yoga training interventions consisted of 6–36 sessions, that were completed 2–6 times per week, over 1–16 weeks. Acute hot yoga increased body temperature and heart rate, but not the energetic demands when compared to other forms of non-heated yoga. Chronic hot yoga appeared to elicit cardiometabolic (e.g. body composition, lipid profiles and macrovascular function) and functional adaptations applicable for health (e.g., bone mineral density, balance and flexibility) as well as physical performance (e.g., submaximal exercise thresholds). Adaptations appear to occur without negatively impacting kidney function or sleep quality across healthy, sedentary and athletic populations. Hot yoga also presents promising, albeit inconclusive findings concerning the alleviation of psychological and affective disorders, and optimising markers of cognitive function. However, caution is advised as case studies report ill-health following hot yoga practice. Some literature lacks rigorous, high-quality experimental designs and sophisticated measurements that allow for mechanistic investigation.

**Conclusion:**

Investigations into hot yoga demonstrate intriguing health and functional benefits. However, claims that hot yoga provides greater health benefits than other forms of yoga or traditional exercise are at present unsubstantiated. Studies describing beneficial effects of hot yoga often do not utilise robust experimental designs or methods that facilitate mechanistic insights. Hot yoga warrants further investigation as a tool to improve health and wellbeing. Researchers should consider the highlighted methodological limitations and recommendations to strengthen experimental work within future research.

## Key Points

1. A single session of hot yoga increases core temperature and heart rate, but not energetic demands, compared to non-heated yoga. However safe practice recommendations (e.g. hydration strategies) should be followed when undertaking this form of physical activity in heat stress, as cases of ill-health and heat illness have been reported.

2. Repeatedly practising hot yoga appears to promote some beneficial adaptations compared to non-exercise and non-heat yoga, including improvements to physiological and functional health (e.g., body composition, balance and flexibility) as well as potential physical performance improvements (e.g., strength). Long-term hot yoga practice may also promote psychological and cognitive function improvements.

3. The hot yoga literature is largely comprised of female participants, which represents a novel area for thermal physiology research to explore. Researchers should however report menstrual cycle status and include non-heated control comparisons to address experimental issues within future research designs.

## Background

Yoga is a widely practised form of physical activity (PA) consisting of transitions between postures, with evidence supporting the potential to enhance individuals’ health and wellbeing across healthy and diseased populations [1]. Hot yoga, however, is a collective term used to classify any form of yoga undertaken in warm to hot ambient conditions (typically within air temperatures of ≥ 25 °C [2]). The overarching premise of hot yoga is that the superimposed heat stress provides a concurrent additional stressor, to potentiate greater beneficial responses to the physical training component of yoga. The traditional form of hot yoga, known as Bikram yoga or also commonly referred to as “26/2”, was developed in the early 1970s and involves a 90 min instructional class, with 26 fixed postures and two breathing exercises (pranayama), which are repeated twice per session in hot conditions (~ 40 °C and ~ 40% relative humidity [RH]). Contemporary forms of hot yoga, including Yin, Hatha, Ashtanga or Vinyasa, that typically allow free selection of yoga poses, are characteristically shorter in duration and similarly occur within a range of warm to hot conditions (~ 25–40 °C). A hot yoga session has been classified according to American College of Sports Medicine criteria [3] as being light- (< 3 metabolic equivalents [METs]) to moderate-intensity exercise (3–6 METs [4]) and may be considered an appropriate form of PA by the World Health Organisation guidelines [5]. Despite this, evidence supporting the use of hot yoga as a PA intervention is lacking relative to other modalities.

Around a decade ago, Hewett et al. [2] critically reviewed Bikram yoga literature (*n* = 10 investigations), providing recommendations for clinical trials and highlighting areas for further investigation. Since then, research studies have investigated hot yoga’s efficacy across physiological, functional and psychological adaptations, demonstrating promising health and performance benefits to a range of populations (e.g. obese, elderly, depressed individuals and athletes). Numerous reviews and meta-analyses have highlighted the potential health benefits associated with non-heated yoga, yet there is limited literature that has systematically reviewed hot yoga research [2]. To this end, evidence has highlighted that hot yoga has the potential to positively modify a range of health outcomes that can be broadly divided into physical (e.g., cardiovascular and metabolic health), functional (e.g. bone mineral density and flexibility), and psychological benefits (e.g., cognitive and functional health, and mood and wellbeing). Given the widespread popularity of hot yoga, there is the potential that scientific evidence is not distinguished from widespread media views (e.g., websites and social media) or commercial yoga studios. Synthesised evidence-based research can help appraise the validity of claims made in these settings, as well as improving the knowledge of teachers and users of hot yoga. However, accessible data and/or a systematic review of the current literature are lacking.

Despite the proposed benefits of hot yoga, it is important to recognise potential risks of heat-related illness (HRI) associated with undertaking this form of PA within heat stress [6]. This may be especially pertinent for those unaware of the signs/symptoms of HRI and/or with compromised health. Thus, developing evidence-based health and safety recommendations is necessary. Furthermore, whilst female-focussed research is lacking across sport, health and exercise science disciplines [7, 8] as well as thermal physiology literature [9, 10], yoga is more commonly practiced amongst females [11]. However, comprehensive reporting of participant characteristics within experimental cohorts participating in hot yoga research is absent. This study therefore aimed to systematically review the literature concerning hot yoga, with particular focus on the acute responses to a single session and appraising health benefits associated with chronic practice across physiological, functional and psychological domains. In addition, we sought to characterise the participants associated with hot yoga research and outline relevant health and safety considerations, with a view to understanding potential risks and providing recommendations for safe and effective teaching and practice.

## Methods

### Search Strategy

This review was conducted in accordance with the Preferred Reporting Items for Systematic reviews and Meta-Analyses (PRISMA) [12]. A search strategy was formulated, consisting of main syntax features medical subject headings (MeSH): (1) “hot yoga” OR “warm yoga” OR “heat yoga” OR “heated yoga”; OR (2) “Bikram yoga” OR “Moksha yoga” OR “Modo Yoga” OR “hot flow yoga” OR “Baptiste Power Vinyasa” OR “CorePower Yoga” OR “Evolation Yoga”. The study selection process was conducted independently in two stages, by two authors (AW, FB). Searches were performed across two main databases, PubMed and SCOPUS, between 1st December 2023 and 1st September 2024. Other sources included reference lists and associated studies outlined in the selected articles.

### Screening and Selection Criteria

Following the searches, studies were initially screened for inclusion, through assessment of title, abstract and access to the full-text article, before removing duplicates. A Population, Intervention, Comparator and Outcome model (PICO) was created and then undertaken to assess the studies’ suitability, with those that did not meet the following criteria being excluded [13]: Population: male or female adults aged ≥ 18 years-old; Intervention: yoga performed in warm to hot ambient conditions (≥ 25 °C) for a minimum duration of 20 min, in a single session (acute) and/or repeated over a minimum of 4 sessions (chronic); Comparator: pre-to-post change in an outcome measure(s) for a single hot yoga session (acute) and/or exercise/training period (chronic); and Outcome measure(s): physiological (e.g., cardiovascular, pulmonary, metabolic, renal, haematological, thermoregulatory, sudomotor), psychological (e.g., psychological well-being, anxiety, depression, stress, distress tolerance and pain perception), functional (e.g., strength, balance, flexibility, well-being factors and sleep) and/or performance (e.g., maximal aerobic capacity, metabolic thresholds, time trial, peak power). Only experimental, observational and interventional research studies and individual case studies written in English were included. Opinion statements, reviews, books (and chapters), theses and surveys were excluded.

### Data Extraction and Analysis

Relevant data were extracted from included studies and separated into two sections: (1) participant characteristics (i.e., number of participants, sex, age, height, mass, experience and aerobic capacity); and (2) yoga interventions (i.e., method, number of sessions, duration, ambient temperature, RH, activity, design, and purpose of study). Outcome measures for acute responses and chronic adaptations to hot yoga were not extracted and only synthesised within the text due to the large variance/disparity in a range of outcome measures. Data were extracted manually into a custom Excel spreadsheet ([AW] Microsoft, USA) and independently cross-checked by four authors (FB, OG, CJ, MJ). Descriptive data are reported as mean ± standard deviation (SD) and frequency or where unattainable, reported as found within the studies (e.g., mean, range).

## Results

### Search Strategy Outcome

**Fig. 1:**
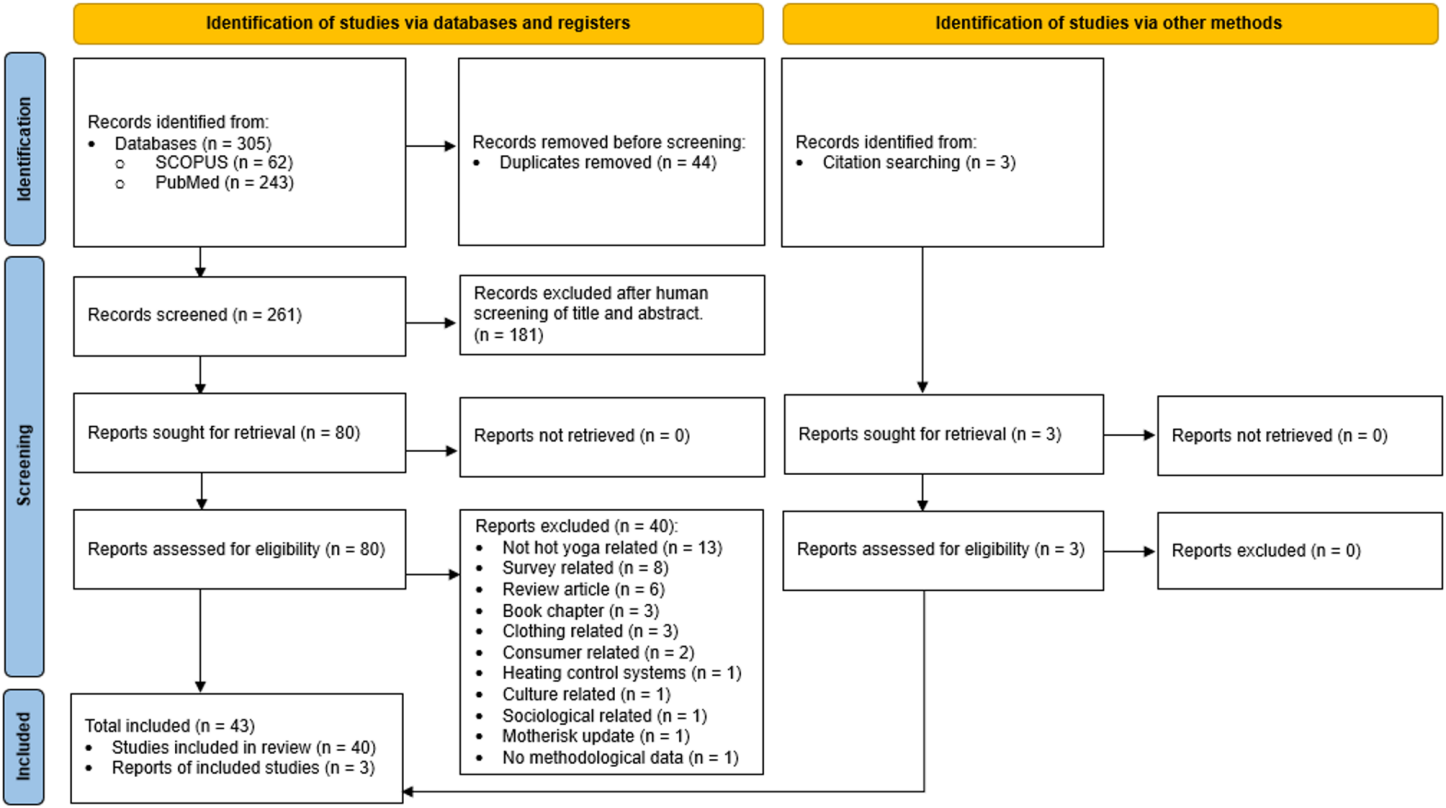
The stages of the selection criteria in accordance with the PRISMA guidelines [12]

Figure 1 illustrates the stages of the selection criteria in accordance with the PRISMA guidelines, which resulted in 43 studies being included in this systematic review.

### Participant Characteristics

In the 43 studies investigating the effects of hot yoga (both acute and chronic), 942 participants were found (20 ± 21 per study [range: 1–137]), of which 76% were female (*n* = 718). However, multiple studies appeared to use a similar cohort of participants across their studies (e.g., “double counting”) and thus, the level of participant independence in the current analysis cannot be ascertained. Nonetheless, participant characteristics and their hot yoga experience are presented in Table 1.

**Table 1:** Hot yoga participant characteristics and experience for acute, chronic and case study literature

Study	Number of participants	Sex (*n*)	V̇O_2max_ (mL.kg^−1^.min^−1^)	Age (years)	Height (cm)	Body mass (kg)	Experience (duration or *n*)	Weekly yoga frequency (*n*)
**Acute**								
Alrefai et al. [14]	5	Female (5)	-	47 ± 5	155.5 ± 3.7	60.4 ± 5.5	3 ± 2 years	4 ± 1
Boyd et al. [15]	14	Male (7) Female (7)	Male: 47.7 ± 2.7 Female: 42.4 ± 2.6	Average: 22 ± 1 Male: 23 ± 1 Female: 21 ± 1	Male: 182.8 ± 2.1 Female: 166.8 ± 2.2	Average: 75.1 ± 4.6 Male: 86.5 ± 5.1 Female: 63.2 ± 4.3	No experience: *n* = 6 1–10 sessions: *n* = 5 90–400 sessions: *n* = 3	-
Dysart and Harden [16]	21	Male (7) Female (13) Unknown (1)	-	48 ± 22	-	-	Yes: *n* = 8 No: *n* = 12 Unknown: *n* = 1	-
Fritz et al. [17]	20	Male (9) Female (11)	-	30 ± 7	-	-	6 ± 5 years	2
Hurtado et al. [18]	16	Female (16)	-	40 ± 8	-	-	-	-
Lambert et al. [19]	16	Male (2) Female (14)	Combined: 36.9 ± 7.6	40 ± 11	162.3 ± 1.9	59.6 ± 6.7	9 ± 1 years	5 ± 3 h per week of total yoga, 3 ± 2 h per week of hot yoga
Mathis et al. [20]	5	Female (5)	-	47 ± 5	155.5 ± 3.7	60.4 ± 5.5	3 ± 2 years	4 ± 1
Pate and Buono [4]	24	Male (5) Female (19)	-	33 ± 13	-	-	-	-
Quandt et al. [6]	20	Male (7) Female (13)	-	Male: 51 ± 8 Female: 42 ± 8	Male: 180.3 ± 6.9 Female:164.3 ± 7.3	Male: 81.6 ± 13.2 Female: 60.8 ± 7.7	-	-
Szabo et al. [21]	53	Male (11) Female (42)	-	39 ± 13	-	66.3 ± 13.4	-	3 ± 1
**Chronic**								
Abel et al. [22]	17	-	-	44 ± 12	165.8 ± 8.0	70.4 ± 13.0	< 3 months: *n* = 17	5 ± 2
	14	-	-	38 ± 9	166.4 ± 7.1	66.95 ± 13.3	1 year: *n* = 14	4 ± 1
Bourbeau et al. [23]	22	Male (11) Female (11)	Average: 44.3 ± 8.1 Male: 48.9 ± 6.7 Female: 39.7 ± 6.7	Average: 26 ± 6 Male: 28 ± 6 Female: 24 ± 51	Average: 169 ± 10 Male: 178 ± 29 Female: 162 ± 7	Average: 70.1 ± 11.8 Male: 77.0 ± 11.3 Female: 63.3 ± 7.8	-	-
Bordman et al. [24]	11	Male (2) Female (9)	-	47 ± 11	-	-	> 7–24 months: *n* = 3 > 24 months: *n* = 8	2–3: 4 4–5: 6 > 5: 1
Flehr et al. [25]	17	Female (17)	-	31 ± 7	166.5 ± 4.4	75.9 ± 13.7	-	1 ± 1
Fritz and O’Connor [26]	16	Female (16)	-	Range: 18–24	-	-	-	-
Hart and Tracy [27]	10	Male (4) Female (6)	-	29 ± 6	170 ± 8.2	73.8 ± 5.6	-	-
Hewett et al. [28]	51	Male (10) Female (41)	Combined: 38.4 ± 7.1	32 ± 9	-	-	Yes: *n* = 14 No: *n* = 37	-
Hewett et al. [29]	29	Male (6) Female (23)	-	38 ± 10	-	86.4 ± 21.2	-	-
Hewett et al. [30]	29	Male (6) Female (23)	-	38 ± 10	-	86.4 ± 21.2	-	-
Hewett et al. [31]	29	Male (6) Female (23)	-	38 ± 10	-	86.4 ± 21.2	-	-
Hopkins et al. [32]	27	Female (27)	-	Range: 25–45	-	-	-	-
Hui et al. [33]	137	Male (30) Female (107)	-	30 ± 8 29 ± 9	-	-	-	-
Hunter et al. [34]	14 (young, lean)	Male (3) Female (11)	-	32 ± 10	167 ± 8	61.5 ± 7.5	-	-
	15 (older, obese)	Male (3) Female (12)	-	46 ± 12	169 ± 7	98.3 ± 16.1	-	-
Hunter et al. [35]	24 (young)	Male (3) Female (21)	-	30 ± 1	167 ± 2	74.7 ± 3.7	-	-
	18 (middle and older aged)	Male (5) Female (13)	-	53 ± 2	169 ± 2	86.7 ± 5.1	-	-
Hunter et al. [36]	21 (normal BMI)	Male (4) Female (17)	-	32 ± 10	166 ± 8	60.8 ± 7.6	-	-
	22 (overweight, obese)	Male (4) Female (18)	-	43 ± 12	168 ± 7	92.4 ± 14.4	-	-
Hunter et al. [37]	17 (young)	Male (3) Female (14)	-	31 ± 2	166 ± 2	72.9 ± 4.2	-	-
	19 (middle and older aged)	Male (6) Female (13)	-	50 ± 2	169 ± 2	85.6 ± 5	-	-
Hunter et al. [38]	19	Male (4) Female (15)	-	47 ± 5	166 ± 7	77.7 ± 19.3	-	-
Hunter et al. [39]	21	Male (5) Female (16)	-	47 ± 5	166 ± 7	77.7 ± 19.3	-	-
Hunter et al. [40]	9	Female	-	Range: 20–56	-	-	-	-
Kudesia and Bianchi [41]	13	Male (7) Female (6)	-	Range: 22–43	-	--	Experienced: *n* = 4 Novice: *n* = 9	-
La Rocque et al. [42]	15	Female (15)	-	34 ± 16	-	-	-	-
Medina et al. [43]	27	Female (27)	-	32 ± 5	-	-	-	-
Nyer et al. [44]	28	Male (8) Female (20)	-	37 ± 14	-	-	-	-
Nyer et al. [45]	33	Male (8) Female (25)	-	32 ± 12	-	-	-	-
Perrotta et al. [46]	10	Female (10)	-	26 ± 3	170 ± 4	65.9 ± 5.4	-	-
Sangiorgio et al. [47]	9	Female (9)	-	51 ± 8	163.4 ± 4.2	74.1 ± 1.9	Minimum 3 years	
Tracy and Hart [48]	10	Male (4) Female (6)	-	29 ± 6	-	-	-	-
**Case Study**								
Boddu et al. [49]	1	Female (1)	-	35	-	-	-	-
Ferrera et al. [50]	1	Male (1)	-	53	-	-	-	-
Lu and Pierre [51]	1	Male (1)	-	33	-	-	-	-
Reynolds et al. [52]	1	Female (1)	-	34	-	-	-	-
Sakurai et al. [53]	1	Female (1)	-	28	-	-	-	-
Takeuchi et al. [54]	1	Female (1)	-	23	-	-	-	-

Note: Data are reported as mean ± SD, range and/or frequency (n). “-“ = data not reported

### Hot Yoga Methods

A summary of hot yoga methods is presented in Table 2. The most common method of yoga was Bikram (74%), followed by generalised hot yoga (19%), Hatha (5%) and then Vinyasa (2%). Most studies reported only ranges and/or the initial prescriptive methodological data, neglecting adherence or session completion rates (Table 2). Thus, we provide herein the data reported within the proposed methods of included studies. Readers are directed to individual studies for further information. Nonetheless, across all the hot yoga studies, a typical session duration ranged 20–90 min and occurred within 30–52 °C and 20–60% RH. Chronic hot yoga interventions consisted of 6–36 sessions, completed 2–6 times per week, over 1–16 weeks (Table 2). Most study designs were experimental (81%), followed by case reports (14%) and longitudinal monitoring (7%). In the acute studies, only 30% (*n* = 3/10) used non-heated yoga as a control condition. Whereas, within chronic studies, 67% (*n* = 18/27) used one or two control groups, ranging from non-heated yoga, high-intensity interval training (HIIT), or aerobic training, as well as non-exercising control groups that maintained routine lifestyle practices or were placed on a “wait list”. Slightly more than half of the chronic studies (56%, *n* = 15/27) employed a randomised controlled trial (RCT) design.

**Table 2:** Hot yoga methods and purpose for acute, chronic and case study literature

Study	Method	Sessions (*n*)	Duration (min)	Temperature (˚C)	Relative humidity (%)	Design	Purpose
**Acute**							
Alrefai et al. [14]	Bikram yoga	1	90	40.6	40	Experimental study without control group	Quantify sweat loss/composition, water consumption and their effect on body fluid volume and electrolyte levels during a single Bikram yoga session.
Boyd et al. [15]	Standardised hot yoga	1	20	35.3 ± 0.8	21 ± 1	Experimental study with non-heated yoga control group	Determine objective and subjective measures of exercise intensity during constant intensity yoga in hot and temperate yoga.
Dysart and Harden [16]	Hatha hot yoga	1	60	33.3	-	Experimental study with non-heated yoga control group	Determine objective measure of exercise intensity between temperature of Hatha yoga.
	Vinyasa hot yoga	1	60	33.3	-		Determine objective measure of exercise intensity between temperature of Vinyasa yoga.
Fritz et al. [17]	Bikram yoga	1	90	40.6	40	Experimental study without control group	Characterise the metabolic, cardiovascular and temperature changes during a session of Bikram yoga.
Hurtado et al. [18]	Bikram yoga	1	90	-	-	Experimental study without control group	Evaluate the physiological responses (blood pressure, heart rate and myocardial oxygen consumption) during a single session of Bikram yoga.
Lambert et al. [19]	Bikram yoga	1	60	40.6	40	Experimental study with non-heated yoga control group	Investigate the acute (single session) physiological effects hot yoga compared to temperate yoga for aerobic intensity, EE, resting haemodynamics, range of movement and biomarkers related to exercise stress and inflammation in experienced yoga practitioners.
Mathis et al. [20]	Bikram yoga	1	90	40.6	40	Experimental study without control group	Determine whether calcium lost through sweat causes a decrease in serum calcium and quantify the amount of calcium lost in sweat during Bikram yoga.
Pate and Buono [4]	Bikram yoga	1	-	-	-	Experimental study without control group	Investigate the EE, HR, sweat rate, and T_CORE_ associated with a single session of Bikram yoga.
Quandt et al. [6]	Bikram yoga	1	90	40.6	40	Experimental study without control group	Examine the HR, T_CORE_ and RPE responses during a single session of Bikram yoga.
Szabo et al. [21]	Bikram yoga	1	90	40.6	-	Experimental study without control group	Investigate the acute (single session) psychological effects of Bikram yoga in context of perceived stress on positive and negative affect and state-anxiety.
**Chronic**							
Abel et al. [22]	Bikram yoga	-	-	-	-	Experimental study without control group	Describe the resting cardiovascular, lung function and aerobic fitness characteristics of long-term Bikram yoga practitioners and examine the relationship between previous Bikram yoga experience and these physiological measures.
Bourbeau et al. [23]	Standardised hot yoga consisting of 24 postures	12 (3 per week, 4 weeks)	75	Range 37–40	40	Experimental RCT study with non-heated yoga control group	Compare cardiovascular, cellular and neural adaptations of hot and temperate yoga over 4 weeks.
Bordman et al. [24]	Hot yoga of any movement	-	60–120	> 35 °C	-	Observational study with non-heated yoga control group	Investigate acute and chronic changes in renal function between practitioners of hot and non-heated yoga.
Flehr et al. [25]	Bikram yoga	24 (3 per week, 8 weeks)	90	40	40	Experimental RCT study with non-heated HIIT control group	Determine the efficacy and assess feasibility of Bikram yoga to improve pain severity and interference in females with persistent pain and a history of trauma
Fritz and O’Connor [26]	Bikram yoga	12 (2 per week, 6 weeks)	90	40.6	-	Experimental RCT study with non-exercising control group	Determine the feasibility of Bikram yoga and effect on executive function.
Hart and Tracy [27]	Bikram yoga	24 (3 per week, 8 weeks)	90	Range 35.0–40.6	60	Experimental study with non-exercising control group	Describe the effects of 8 weeks of Bikram yoga on the strength and steadiness of proximal muscles.
Hewett et al. [28]	Bikram yoga	24 (3 per week, 8 weeks)	90	40.6	40	Experimental study without group	Investigate the effects of 8 weeks of Bikram yoga on mindfulness, perceived stress and several components of physical fitness; and examine the relationships between intervention-related changes in mindfulness with measures of perceived stress and physical fitness.
Hewett et al. [29]	Bikram yoga	48–80 (3–5 per week, 16 weeks)	90	40.6	40	Experimental RCT study with non-exercising control group	Investigate the effects of 16 weeks of Bikram yoga on perceived stress, increase general self-efficacy and improve all domains of health-related quality of life compared to participants randomised to a no-treatment control group.
Hewett et al. [30]	Bikram yoga	48–80 (3–5 per week, 16 weeks)	90	40.6	40	Experimental RCT study with non-exercising group	Investigate the effect of 16 weeks of Bikram yoga on cardiovascular function and disease risk factors in a population of stressed and sedentary adults.
Hewett et al. [31]	Bikram yoga	48–80 (3–5 per week, 16 weeks)	90	40.6	40	Experimental RCT study with non-exercising group	Investigate factors that predicted and acted as perceived barriers to adherence to Bikram yoga over 16 weeks in stressed and sedentary adults.
Hopkins et al. [32]	Bikram yoga	16 (2 per week, 8 weeks)	90	40.0	-	Experimental RCT study with non-exercising group	Examine the effects of 8 weeks of heated hatha yoga on cortisol reactivity to stress and examine intervention effects on self-report measures of binge eating and affective eating.
Hui et al. [33]	Hot yoga	24 (4 per week, 6 weeks)	90	-	-	Experimental RCT study with non-exercising group	Investigate the impact of 6 weeks of hot yoga on wellbeing.
Hunter et al. [34]	Bikram yoga	24 (3 per week, 8 weeks)	90	40.6	Range 40–60	Experimental study without control group	Determine the effect of 8 weeks of Bikram yoga on glucose tolerance in young lean adults and older obese adults.
Hunter et al. [35]	Bikram yoga	24 (3 per week, 8 weeks)	90	40.6	Range 40–60	Experimental study without control group	Determine the effect of 8 weeks of Bikram yoga on arterial stiffness and insulin resistance in young and older adults.
Hunter et al. [36]	Bikram yoga	24 (3 per week, 8 weeks)	90	40.6	Range 40–60	Experimental study without control group	Investigate the impact of 8 weeks of Bikram yoga on arterial stiffness in normal and overweight/obese adults.
Hunter et al. [37]	Bikram yoga	24 (3 per week, 8 weeks)	90	41.0	Range 40–60	Experimental study without control group	Determine the effects of 8 weeks of Bikram yoga on endothelial function in young and old adults.
Hunter et al. [38]	Bikram yoga	36 (3 per week, 12 weeks)	90	40.6	-	Experimental RCT study with non-heated yoga and non-exercising control groups	Determine the effects of 12 weeks of Bikram yoga practised in standard heated and non-heated environmental conditions on endothelium-dependent vasodilatation in healthy, middle-aged adults
Hunter et al. [39]	Bikram yoga	36 (3 per week, 12 weeks)	90	40.5	-	Experimental RCT study with non-heated yoga and non-exercising control groups	Investigate the effects of 12 weeks of Bikram yoga on arterial stiffness in middle-aged adults and to explore the potential added effects of environmental temperature on this measure.
Hunter et al. [40]	Hot yoga	20 (5 per week, 4 weeks)	45	52.0	-	Experimental RCT study without non-heated yoga control group	Examine whether 4 weeks of hot yoga could attenuate sodium-induced pressor responses and endothelial dysfunction in Black females.
Kudesia and Bianchi [41]	Bikram yoga	2–12 over 2 weeks	-	-	-	Experimental study with non-heated yoga control days	Investigate the feasibility of using a simple home monitor to characterize sleep architecture in a low-constraint setting, and to determine if 2 weeks of Bikram Yoga acutely affects the sleep architecture of healthy young adults.
La Rocque et al. [42]	Bikram yoga	16 (2 per week, 8 weeks)	90	~ 40	40	Experimental RCT study with aerobic exercise and non-exercising control groups	Examine the efficacy of 8 weeks of Bikram yoga in unipolar depressed females.
Medina et al. [43]	Hatha yoga Bikram yoga	16 (2 per week, 8 weeks)	90	40.0	-	Experimental RCT study with non-exercising control group	Investigate whether an 8 week hot hatha yoga intervention can enhance distress tolerance.
Nyer et al. [44]	Bikram yoga	16 (2 per week, 8 weeks)	90	40.6	40	Experimental study without control group	Investigate the acceptability and feasibility of 8 weeks of heated yoga as a treatment of depression and explore its association with depressive symptoms.
Nyer et al. [45]	Bikram yoga	16 (≥ 2 per week, 8 weeks)	90	40.6	-	Experimental RCT study with non-exercising control group	Evaluate feasibility, acceptability, and preliminary efficacy of 6 weeks of heated yoga to treat moderate-to-severe depression.
Perrotta et al. [46]	Hot yoga	6	60	30.0 ± 1.8	48 ± 9	Experimental study without control group	Investigate the effectiveness of six hot yoga sessions for inducing hypervolemia and augmenting cardiovascular performance within a national field hockey team.
Sangiorgio et al. [47]	Bikram yoga	Taught and participated 3 sessions per week over 5 years	90	-	-	Longitudinal study without control group	Evaluate bone mineral content of the proximal femur and lumbar spine following 5 years of yoga practice and teaching.
Tracy and Hart [48]	Bikram yoga	24 (3 per week, 8 weeks)	90	Range: 35–41	60	Experimental RCT study with non-exercising control group	Examine the effect of 8 weeks of Bikram yoga on general physical fitness.
**Case Study**							
Boddu et al. [49]	Hot yoga	1	-	-	-	Case study	Case report detailing sudden cardiac arrest from heat stroke during hot yoga.
Ferrera et al. [50]	Bikram yoga	1	90	-	-	Case study	Report acute coronary syndrome during Bikram yoga.
Lu and Pierre [51]	Bikram yoga	-	-	-	-	Case study	Report psychosis event during Bikram yoga.
Reynolds et al. [52]	Bikram yoga	1	90	40.6	-	Case study	Report exercise associated hyponatraemia leading to tonic-clonic seizure during a single session of Bikram yoga.
Sakurai et al. [53]	Hatha yoga	12 (over 8 weeks)	90	40.6	-	Case study	Report the efficacy of 8 weeks of Hatha yoga in a patient with a history of treatment-resistant depression.
Takeuchi et al. [54]	Hot yoga	-	-	-	-	Case study	Report photo urticaria in a hot yoga instructor.

Note: HIIT = High-intensity interval training, RCT = randomised controlled trials, EE = energy expenditure, HR = heart rate, T_CORE_ = core temperature, and RPE = rating of perceived exertion. Data are reported as mean ± SD, reported mean, range and/or frequency (n). “-“ = data not reported

The following sections of this systematic review outline: (1) the acute responses to a single session of hot yoga; (2) the physiological, functional and psychological responses and adaptations following repeated hot yoga; and (3) single participant case studies associated with hot yoga.

### Acute Responses To Hot Yoga

Ten studies have investigated the acute responses to hot yoga. Within these, physiological strain during hot yoga appears modest, with exercise intensity having been classified as light-to-moderate [4]. Many yoga postures require forceful, upper- and lower-limb isometric contractions. During Bikram yoga, this has been shown to elicit an oxygen uptake (V̇O_2_) of 6–13 mL.kg^−1^.min^−1^ (mean: 9.5 ± 1.9 mL.kg^−1^.min^−1^ [4]), which corresponds to 25–31% V̇O_2max_ [4, 15, 19]. Greater intensity has been observed during standing (14.2 ± 1.6 mL.kg^−1^.min^−1^) compared to floor postures (11.4 ± 1.0 mL.kg^−1^.min^−1^), with similarly higher heart rate (HR: +21 b.min^−1^ [17]) during standing postures. Energy expenditure (EE) reported during 60 min of Bikram yoga has been reported as 156 ± 7 [19], up to 286 ± 72 kcal.hr^−1^ (range of individual responses: 179–478 kcal.hr^−1^, [4]). A higher total EE of 378 ± 78 kcal has been recorded during longer 90 min sessions (range: 278–541 kcal, female: 333 ± 46 kcal, male: 459 ± 55 kcal [17]). Some of the variance in EE may be explained by yoga competency, with higher observed EE (4.7 ± 0.8 vs. 3.7 ± 0.5 kcal.kg^−1^), HR (86 ± 5 vs. 72 ± 10% HR_max_), oral temperature (+ 1.0 ± 0.8 vs. +0.6 ± 0.7 °C) and sweat response (1.1 ± 0.5 vs. 0.5 ± 0.2 kg.hr^−1^) in experienced, compared with novice participants, respectively [4]. This may reflect experienced hot yoga users achieving a larger range of motion (ROM) and greater muscle mass recruitment [4]. Further variance in physiological responses may reflect individual characteristics, such as age, clothing, muscle mass and body surface area, which influence human heat exchange [55]. Session design (i.e., floor vs. standing postures) and tempo of activity (i.e., exercise intensity) are also contributing factors to variable responses within the literature. Clear evidence is lacking that exercise intensity and EE during hot yoga differs to non-heated yoga [15]. Notably, hot yoga does not appear to increase joint ROM above other forms of yoga, considering only one of thirteen ROM measures was found to be different [19]. Lambert et al. [19] suggested the demands of the predominantly static poses appear insufficient to elevate EE during hot yoga. However, the mechanistic basis for greater EE during low-intensity activity is unclear. A greater energetic cost of equivalent exercise in the heat versus temperate conditions is not routinely observed [56]). Hyperventilation may be one mechanism eliciting enhanced EE demands within heat stress [57]. However this is unlikely to occur during yoga, and it should also be noted that a warmer muscle displays greater efficiency (i.e., the level of force for a given quantity of neural drive [58, 59]). Overall, hot yoga does not consistently or meaningfully appear to elevate acute EE or V̇O_2_, although perceived exertion may be greater [15].

Peak core temperatures (T_CORE_) during 90 min of Bikram yoga have ranged between 38.2 and 40.1 °C (females: 38.9 ± 0.5 °C, males: 39.6 ± 0.4 °C [6]). Fritz et al. [17] reported that peak T_CORE_ (38.2 ± 0.3 °C, + 0.8 °C from baseline), typically occurred at the end of the standing postures (~ 50 min). Thereafter, activity may be thermally compensable (i.e., a steady state T_CORE_ can be achieved), although this finding is not universal [6]. Elevated T_CORE_ may contribute to seemingly altered substrate utilisation, through a lower respiratory exchange ratio (RER: -0.06 A.U.) and indicated inflammatory response (serum interleukin-6: increased ~ + 2.0 pg.dL^− 1^ at 41.5 °C vs. ~+0.2 pg.dL^− 1^ at 23.5 °C [19]). Quandt et al. [6] reported average HR to be 75% of predicted maximum (females: 72 ± 8%, males: 80 ± 8%), with peak HR of 85 ± 6% and 92 ± 8% HR_max_ for females and males, respectively. Sweat losses from 90 min of Bikram yoga were 1.5 ± 0.7 L, with 0.4 ± 0.2 L of water consumed (~ 25% replenishment) [14]. Despite a 9.7% decrease in extracellular fluid, there were no changes in serum sodium or osmolality, indicating a shift in fluid compartments, rather than dehydration per se. This would likely be restored within ~ 24 h with adequate rehydration or, avoided with 45% replenishment during the session [14].

Acute hot yoga may reportedly decrease acute state-anxiety and negative affect (i.e., negative emotional states) and increase positive affect [21], similar to strenuous exercise (e.g. spin class) [60] or other forms of non-heated yoga (e.g., Vinyasa [61], Hatha [62]). Acute psychological changes demonstrate associations with those experiencing stress in daily life [21]. However, it should be acknowledged that evidence pertaining to psychological effects is limited. Moreover, individuals often engage in hot yoga with an expectation of perceived benefits, meaning a placebo effect cannot be discounted. In summary, acute hot yoga may increase T_CORE_, HR and sweat rate, but not the energetic demands of the activity compared to other forms of yoga, performed in cooler environments. However, acute hot yoga does influence the psychological experience, and participants’ experience may further modify the physiological responses.

### Cardiovascular, Metabolic and Renal Responses and Adaptations To Hot Yoga

Fourteen studies examined the efficacy of chronic hot yoga on cardiovascular, metabolic and/or kidney function outcomes. In one of the first studies examining these outcome measures following 8 weeks (3 sessions per week) of Bikram yoga, reductions in body mass (-1.2 kg) and body mass index (BMI: -0.6 kg.m^2^) in obese participants were reported [34]. Subsequent studies reported improvements in sit and reach flexibility (+ 33%), total (-16 mg.dL^− 1^) and low-density lipoprotein (LDL) cholesterol (-18 mg.dL^− 1^), insulin concentration (-7 µIU.mL^− 1^) and decreased homeostatic model assessment for insulin resistance (HOMA-IR: -2.6 A.U.) in older adults, whereas younger adults reported increased flexibility (+ 16%), and reduced total (-13 mg.dL^− 1^) and high-density lipoprotein (HDL) cholesterol (-6 mg.dL^− 1^) [35]. Body mass, body fat percentage, blood pressure, fasting blood glucose and triglyceride concentrations did not significantly change following Bikram yoga in either group [35]. These data indicate that Bikram yoga may positively enhance the metabolic profile of younger and older adults, and body composition of obese individuals, although additional research is warranted to support these findings.

In addition to these metabolic responses, hot yoga has also been reported to induce several positive cardiovascular adaptations. Hunter et al. [35] reported functional cardiovascular adaptations, such as increased carotid artery compliance (+ 0.02 mm^2^.mmHg) and β-stiffness (~ 1 U) in young but not older adults. Regrettably this study was unable to identify why adaptation was not possible with the older cohort, despite inferior cardiovascular profiles at baseline (thus possessing a greater potential to adapt relative to the young). Reduced arterial stiffness (i.e., decreases in brachial-ankle pulse wave velocity [PWV]) of -45 cm.s^− 1^ has been evidenced in overweight/obese adults, but not adults with normal BMI undertaking Bikram yoga [36]. Additional macrovascular adaptations following hot yoga have also been identified using brachial artery flow mediated dilatation (FMD), with significant increases in FMD (+ 1.4%) in middle-aged/older adults, yet no response occurring in a young cohort [37]. Interestingly, hot yoga increased FMD during a sodium loading challenge relative to the pre-intervention state, where FMD decreased [40]. This post-intervention response occurred in the absence of changes in blood pressure.

More recently, hot yoga (75 min, 3 sessions per week for 4 weeks) has been used to increase V̇O_2max_ by 7% in healthy but untrained participants (3.22 ± 0.76 vs. 3.46 ± 0.79 L.min^−1^). Thermotolerance, as quantified by a 23% increase in intracellular heat shock protein (HSP) 70, was also found following hot, but not non-heated yoga [23]. The increase in V̇O_2max_ can be viewed as a positive outcome given the importance of the variable in both cardiorespiratory fitness and morbidity and mortality contexts [63]. The relevance of enhanced HSP is presently less clear, but this molecular adaptation has the potential to positively enhance several disease states. A number of studies however have reported no beneficial outcomes to cardiovascular and metabolic health following hot yoga. No changes in HR variability or a panel of cardiovascular risk factors (i.e., HR and blood pressure, lipid profile and fasted glucose, body mass and body composition) were reported in stressed and sedentary adults undertaking 16 weeks (3–5 sessions per week) of Bikram yoga [30]. Additionally, brachial-ankle PWV was unaltered by hot and non-heated Bikram yoga (12 weeks, 3 sessions per week) in sedentary older adults [39]. It is noteworthy that one experiment has also identified that non-heated yoga, but not hot yoga (both 12 weeks, 3 sessions per week), increased brachial artery FMD in healthy older adults [38]. This may imply that yoga postures alone, rather than in conjunction with heat stress, are the more potent stimulus for vascular adaptation.

Contrary to acute EE differences, hot yoga experience level does not appear to confer additional benefit, as practitioners with ~ 4 years of training did not demonstrate different cardiopulmonary outcomes or have a greater V̇O_2max_ than novices of ~ 4 weeks of training [22]. Furthermore, it has been reported that 8 weeks (2 sessions per week) of Bikram yoga reduced cortisol reactivity to stress as well as affective eating among females intentionally restricting food intake to control weight [31]. Finally, within a single observational study, no significant effects were found when investigating kidney function acutely or over a 12 month period within practitioners undertaking hot yoga (2–5 sessions per week), nor were differences found between hot and non-heated yoga during the same timeframe [24].

Overall, whilst acknowledging the null effects reported, hot yoga appears to elicit some cardiovascular and metabolic adaptations in young, lean adults, with positive modifications to body mass, lipid profile, macrovascular function, and aerobic capacity also observable in some older and obese cohorts, without affecting kidney function. These outcomes are not ubiquitous across experimental studies, with a need for more robust experimental designs (e.g. matched exercise controls and RCT) to be implemented to generate more compelling data supporting the advantages of hot yoga, or otherwise.

### Functional Health, Performance and Sleep Responses and Adaptations To Hot Yoga

Among healthy adults and populations at risk of chronic disease (e.g., obesity), hot/Bikram yoga may provide acute and chronic functional health benefits (e.g., improved physical fitness, sleep quality, bone mineral density [BMD]). This premise is based on improved blood flow [34, 35, 46] as well as greater focus and awareness on breathing [28], supposed to maximise mindfulness, calmness and determination. In reference to physical fitness, 8 weeks (3 sessions per week) of Bikram yoga significantly improved strength (isometric deadlift: +13% for hot yoga vs. -4% for non-exercising control, isometric maximal voluntary contraction: +14% vs. -10%), balance (single-leg balance test: +228% vs. +29%) and flexibility (range of motion of lower back: +33% vs. -1%, hamstrings and shoulders: +17% vs. +3%) compared to non-exercising control groups [27, 48]. No changes however were observed in upper-body strength (isometric handgrip strength, elbow flexor strength and steadiness) and cardiovascular measures or maximal aerobic fitness, despite a slight decrease in body fat [27, 48]. These RCTs support previous studies reporting improved flexibility (+ 16–33% [28, 34]) and balance (+ 73% [28]) but not body composition [28, 34, 35], resting hemodynamic, pulmonary function or cardiorespiratory fitness [22].

Hot yoga has been utilised as an intervention purported to provide ergogenic benefit for sports performance. Ten elite female field hockey players completed six, 60 min hot yoga sessions prior to a national training camp [46]. Following the intervention, moderate hypervolaemia (via plasma volume [PV] expansion: +5.0 ± 6.4%) occurred alongside small improvements in running speed at the first (+ 0.3 km.hr^− 1^) and second (+ 0.5 km.hr^− 1^) ventilatory threshold [46]. Trivial effects were also found in maximal aerobic fitness (+ 0.4%) and running time to exhaustion (+ 0.8%), as well as small meaningful improvements in the RER ratio during high-intensity exercise (+ 1%) [46]. The long-term practice of Bikram yoga (taught and participated 3 sessions per week over 5 years) has also been shown to increase BMD (femoral neck: +7%, total hip: +2%, lumbar spine: +1%) in premenopausal female Bikram yoga teachers [47]. While total sleep time, sleep latency and stages of sleep were unaffected by Bikram yoga [41], the beneficial effect on physical fitness and health indicators are dependent on adherence, which has been associated with variable predictors (e.g., older age, less pain, fewer physical limitations, poorer blood lipid profile and higher HR variability) and barriers to participation (e.g., lack of enjoyment, time commitment and adverse events) among stressed and sedentary participants [31].

Overall, hot yoga appears to improve functional health (e.g., BMD, balance and flexibility) as well as some indices of physical performance (e.g., submaximal exercise thresholds) without negatively impacting sleep quality across healthy, sedentary and elite athlete populations.

### Psychological, Cognitive Function and Wellbeing Responses and Adaptations To Hot Yoga

Most studies included in this systematic review assessed the efficacy of hot yoga on psychological and cognitive outcomes (*n* = 9), exploring chronic (*n* = 7) rather than acute (*n* = 2) effects. The limited work investigating the effect of acute interventions is varied in focus (i.e., anxiety [50%] and psychological well-being [50%]), whereas chronic interventions have investigated affective disorders, including depression (43%), stress (14%) and anxiety (14%). In the remaining two articles, the authors examined the effect of chronic hot yoga on distress tolerance (14%) and perceptions of pain (14%). The chronic effects of 8–16 weeks of hot/Bikram yoga on psychological variables are indicative of healthy functioning, psychological wellbeing and cognitive function, providing promising albeit inconclusive evidence. Six weeks (4 sessions per week) of Bikram yoga demonstrated beneficial effects on a range of “wellbeing” outcome measures, with reported improvements across life satisfaction, general health, mindfulness, peace of mind, and eudaimonic wellbeing, but not flourishing [33]. For psychological outcomes, the limited evidence concerns emotional responses to pain, elevating distress tolerance and managing affective disorders, which yield mixed findings. Medina et al. [43] concluded that 8 weeks of twice-weekly Bikram yoga improved distress tolerance and associated affective experiences. Flehr et al. [25] found 8 weeks (3 sessions a week) of Bikram yoga yielded significant reductions in pain indexed through increased physical functioning (80.9 vs. 69.0 A.U.) and improved mental health (63.9 vs. 49.4 A.U.) compared to a high-intensity exercise training group after controlling for baseline physical functioning and mental health indices as a covariate in data analysis.

Research has also examined the efficacy of Bikram yoga compared to control conditions in individuals with mild, moderate and severe affective disorders (such as stress and depression), and reported attenuations in the severity of these characteristics [28–30, 42, 44, 45]. Following a 16 week (3–5 sessions per week) parallel-arm RCT, Bikram yoga resulted in significant attenuations in perceived stress (20.7 A.U. at baseline vs. 12.9 A.U. post-intervention) and alleviated general self-efficacy (29.5 A.U. at baseline vs. 32.6 A.U. post-intervention) in adults with mild to moderate stress compared to a non-treatment control group [28, 29]. A shorter 8 week (≥ 2 sessions per week) Bikram yoga RCT in adults with moderate-severe depression also resulted in significantly greater reductions in depression (36.9 A.U. at baseline vs. 19.9 A.U. post-intervention) compared to a waiting-list control group, although Bikram yoga was not associated with attenuated consumption of anti-depressant medication [45]. Findings also appear to hold true following similar methods in individuals with milder cases of depression [44]; however, inadequate experimental controls mean conclusions concerning the efficacy of Bikram yoga for milder depressive states are tenuous and require further investigation. At present, the limited literature examining the efficacy of Bikram yoga on healthy functioning and psychological wellbeing demonstrates encouraging findings, but requires further research with suitable experimental control, compared against other relevant alternative treatment methods (e.g., aerobic exercise such as jogging or cycling). Indeed, one study [42] compared the efficacy of Bikram yoga alongside the effects of aerobic exercise on depressive symptoms in females and found both interventions held similar descriptive efficacy. Here, La Rocque et al. [42] assigned 53 females with unipolar depressive disorder to one of three conditions: intention-to-treat, Bikram yoga or aerobic exercise. The findings showed similar clinically significant reductions in depression symptoms in both the Bikram yoga (12.9 A.U. at baseline vs. 6.7 A.U. post-intervention) and aerobic exercise groups (11.8 A.U. at baseline vs. 4.8 A.U. post-intervention). Consequently, Bikram yoga twice a week for 8 weeks appears equally efficient as, but no better than, traditional aerobic exercise in attenuating depressive symptoms in a clinically depressed population.

In comparison to research investigating the efficacy of Bikram yoga on affective psychological response, research investigating the effect of Bikram yoga on markers indicative of cognitive function are in their infancy. To date and to the best of the authors’ knowledge, only one study has assessed the efficacy of Bikram yoga on markers of cognitive function. Fritz and O’Connor [26] recruited 32 females with attention deficit hyperactivity disorder (ADHD) and asked half to complete 6 weeks (twice a week) of Bikram yoga. No changes in inhibitory control, cognitive flexibility and working memory were found compared to a wait-list control group; however, direct and conceptual replication is required for stronger assertions concerning the utility of Bikram yoga on cognitive variables.

Taken together, on a psychological and cognitive level and within a limited evidence base, Bikram yoga presents promising, yet inconclusive findings concerning its utility in alleviating psychological and affective disorders and optimising markers of cognitive functions. However, sustained research efforts would benefit from ensuring adequate experimental controls and from testing the effects of Bikram yoga on psychological and cognitive variables in comparison to other alternative exercise treatment methods.

### Case Studies Related To Hot Yoga

As outlined by Racinais et al. [64] and Gibson et al. [65], PA in heat stress presents an increased risk of health complications and performance impairments to end users. Whilst there are limited reports of widespread illness and/or injury incidence attributed to hot yoga practice, there are several case studies which outline reports of ill-health observed during and/or following this type of exercise. Cardiac issues have been reported by way of sudden cardiac arrest during hot yoga in a 35 year-old female [49], with authors suggesting the patient may have experienced a compromised ejection fraction due to post-arrest myocardial dysfunction and hyperthermic cardiac injury. Whilst T_CORE_ was not recorded until 80 min after the initiation of cardiopulmonary resuscitation (36.6 °C), it is suggested the patient likely had a higher T_CORE_ at the onset of cardiac arrect, which then decreased. The report also highlights the potential for some individuals to experience heat stroke symptoms, even when T_CORE_ is below 40 °C.

Acute myocardial infarction has also been observed within a 53 year-old male during Bikram yoga [50]. Exercise-associated hyponatremia (EAH) has been reported in a 34 year-old female following the consumption of 3.5 L of water after her first Bikram yoga session (90 min at 40.6 °C [52]). The patient arrived at an emergency department with signs/symptoms of HRI and EAH (e.g., breathlessness, muscle cramps, nausea and general malaise) before experiencing a tonic-clonic seizure, which resulted in transfer to the intensive care unit for intubation, before full recovery and discharge 5 days later. Dermatological issues (e.g., itching and weals on the neck and face) have been reported in a 23 year-old female hot yoga instructor, whereby photo urticaria (i.e., a type of urticaria in response to exposure of various wavelengths of light) was diagnosed and contributed to by the repetitive exposure to red-light sources (633 nm wavelength light) during modified hot yoga with “*light treatment*” for de-aging purposes [54].

Finally, there is a report of a 33 year-old male experiencing psychosis (e.g., auditory and visual hallucinations, and paranoia) during a Bikram yoga instructors’ training program, which lasted several days and required hospital admission [51]. In contrast to these negative consequences of hot yoga, one case study outlined the benefits of reducing depressive symptoms following 8 weeks of Bikram yoga (12 sessions, 90 min, 40.6 °C) in a 28 year-old female with major depressive disorder [53]. Whilst these case studies report individual findings, it is important to recognise the potential side effects of participating in hot yoga (as discussed below for HRI purposes) as well as understanding the range of contributing factors to cardiovascular, fluid/electrolyte balance and dermatological issues (e.g., health status, underlying issues, diet, education).

## Discussion

The primary aim of this study was to systematically review the literature concerning hot yoga, with particular focus on both the acute responses to a single hot yoga session and chronic adaptations of repeatedly practising hot yoga. The systematic review identified 43 studies that investigated the effects of hot yoga and included 942 participants, 76% of whom were female.

### Acute Responses To Hot Yoga

A single session of hot yoga corresponds to an exercise intensity of ~ 25–31% of V̇O_2max_, with an EE ranging from 156 to 478 to 278–541 kcal for 60 and 90 min durations, respectively [4, 15, 17, 19). These outcomes do not appear to differ from non-heated yoga. Nonetheless, Bikram yoga increases both T_CORE_ (females: ~38.9 °C, males: ~39.6 °C) and HR (females: ~85%, males: ~92% HR_max_ [6]), whilst inducing sweat losses of ~ 1.5 L [14]. The variances within physiological responses may reflect an individual’s level of experience/characteristics and/or session design/intensity. Furthermore, a single session of hot yoga may decrease acute state-anxiety and negative emotional states, as well as increasing positive affect, similarly shown following strenuous exercise or other forms of yoga [21, 61, 62]. The potential for an acute placebo effect is also emphasised, as individuals may engage in hot yoga with prior expectations of its “perceived” benefits. Based upon these acute responses to hot yoga, repeatedly undertaking this form of PA in heat stress is likely to induce an array of beneficial health, functional and performance adaptations, as discussed below.

### Chronic Responses To Hot Yoga

At the chronic level, the most common methods were Bikram and generalised hot yoga, which widely varied in weekly frequency, duration and ambient conditions. The efficacy of hot yoga to induce beneficial physiological and functional adaptations has been examined across different cohorts with a comprehensive array of variables (including cardiovascular, pulmonary, metabolic, haematological, renal, thermoregulatory and sudomotor function, as well as strength, balance, flexibility, BMD and athletic performance). Data support the efficacy of hot yoga to enhance the metabolic profile of younger and older adults [34]. Macrovascular adaptations have also been observed [35], with one study reporting increased cardiorespiratory fitness (e.g., V̇O_2max_ [23]). However, there are limited improvements observed in body composition [28, 34, 35] and cardiorespiratory fitness measures elsewhere in the literature [22, 27, 48]. Nonetheless, compared to non-exercise control conditions, longer-term practice of hot yoga (> 6 weeks) has been found to significantly improve participants’ strength, balance and flexibility [27, 48], with concomitant improvements in BMD [47] and reduced cortisol reactivity to stress [32]. Furthermore, sleep quality/pattern appears unaffected by hot yoga [41], which is advantageous for athletic populations who prioritise sleep/recovery. Six daily hot yoga sessions demonstrated increased running speed at submaximal ventilatory thresholds within elite populations, which is likely mediated by heat acclimation induced physiological processes (e.g., PV expansion [46]). A critique of the existing literature in this domain is that hot yoga is rarely compared to other exercise or passive heating interventions (heat/thermal-therapy [66, 67]), both of which can elicit comparable adaptations, independently. Indeed, one study reported that non-heated yoga induced greater macrovascular adaptations than those observed following hot yoga [39]. This suggests that more comprehensive experimental efforts are required to fully elucidate the combined stressors associated with hot yoga against the independent effects of yoga exercise and thermal strain. In addition, comparing adaptations arising from hot yoga against other common exercise interventions requiring similar time commitments will assist individuals with making decisions regarding PA choices. Aside of the concerns regarding adherence and barriers to hot yoga (as outlined above), pre-existing conditions and individual training responses likely contribute to the limited evidence-based conclusions that can be drawn [68]. This highlights the need for further intensity-matched controlled studies, to elucidate whether the additional heat stress during yoga plays a key role in physical and functional health and wellbeing.

**Fig. 2:**
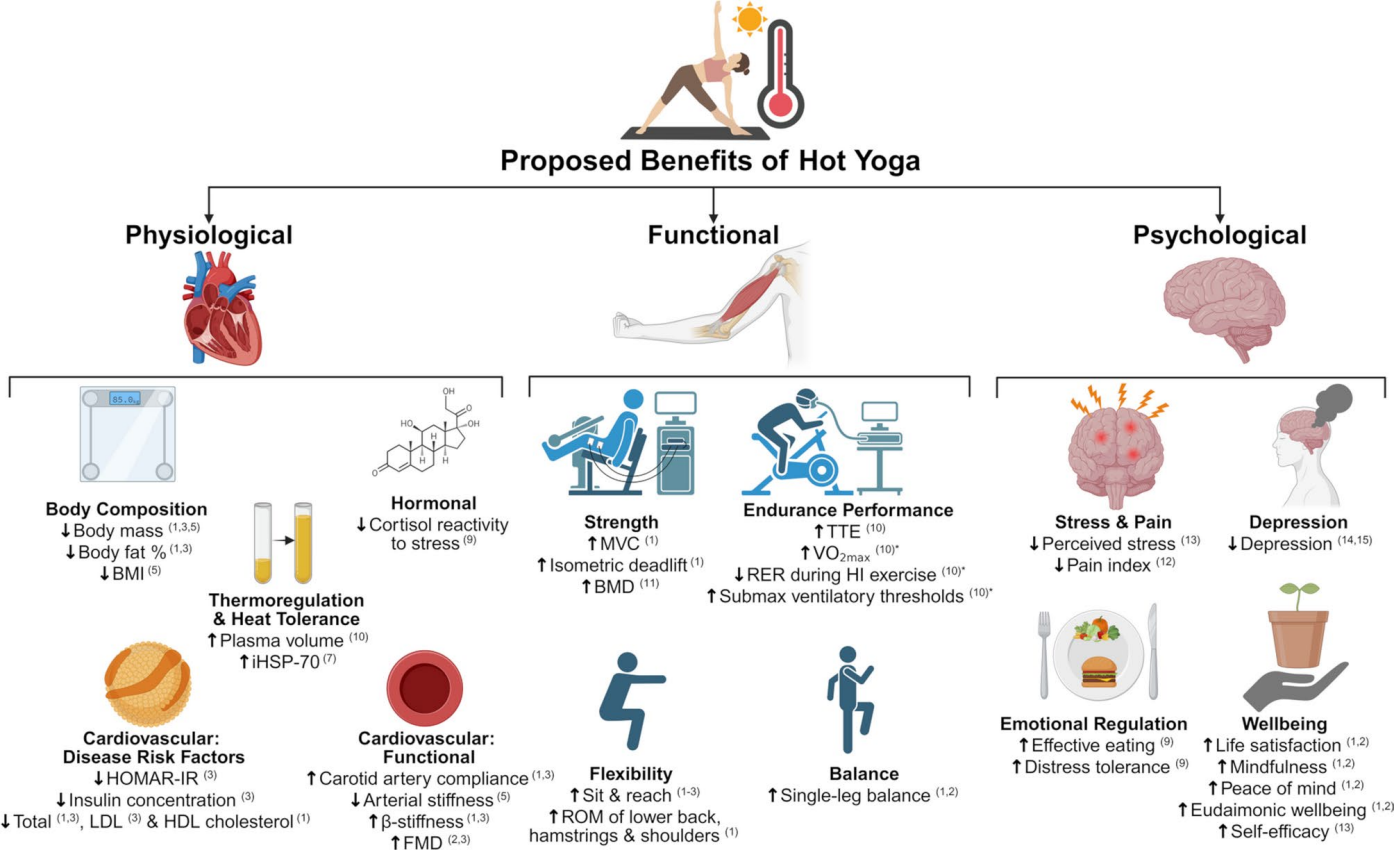
Proposed physiological, functional and psychological benefits of hot yoga. Populations: 1 = Young (18–30 year-old), 2 = Middle-aged (31–55 year-old), 3 = Older (> 55 year-old), 4 = Normal BMI, 5 = Overweight/Obese, 6 = Sedentary & Older, 7 = Healthy, untrained, 8 = Stressed & Sedentary, 9 = Females high in elevated levels of perceived stress, emotional eating & dietary restraint, 10 = Elite Athletes, 11 = Premenopausal females, 12 = Young-middle-aged females with a persistent pain condition and self-reported history of trauma, 13 = Mild to moderate stress, 14 = Moderate-severe depression, and 15 = Unipolar depressive disorder. Note: * links to “physiological” benefits, BMI = body mass index, FMD = flow mediated dilation, iHSP = intracellular heat shock proteins, LDL = low-density lipoprotein, HDL = high-density lipoprotein, HI = high-intensity, HOMA-IR = homeostasis model assessment for insulin resistance, BMD = bone mineral density, MVC = maximal voluntary contraction, V̇O_2max_ = maximal volume of oxygen uptake, TTE = time to exhaustion, RER = respiratory exchange ratio, and ROM = range of motion (note: figure created using BioRender.com, yoga vector created using www.freepik.com)

All forms of hot yoga currently show apparent promising, yet inconclusive, effects on psychological wellbeing and cognitive function. Bikram yoga demonstrates improvements across a range of wellbeing measures (including life satisfaction mindfulness, peace of mind, and eudaimonic wellbeing [33]) and attenuates affective disorder symptoms (ranging across mild [29, 30], moderate [44] and severe [45]), whilst also promoting increased distress tolerance [43] and reducing pain. Together, these findings may provide facilitative adaptations to mental health [25]. However, literature assessing the efficacy of hot yoga on psychological variables may be restricted by inadequate experimental controls and will benefit from comparison to additional or concurrent traditional treatment modalities (e.g., aerobic exercise [42]). Existing data tentatively suggest hot yoga to be as effective, but not superior to traditional treatments. To date, interventions testing the efficacy of hot yoga on cognitive function are in their infancy, with only one known study [26] concluding no effect of 6 weeks of Bikram yoga on inhibitory control, cognitive flexibility and working memory, although direct and conceptual replication attempts are required. Collectively, and in the presence of considerable variability in a small evidence pool, research may benefit from a consistent approach to identify the most efficient, advantageous hot yoga intervention method and duration on psychological and cognitive outcomes, e.g., standardised hot yoga protocols, comprehensive participant data, validated outcome measures, robust experimental designs, adherence monitoring, consideration of inter-individual differences, and transparent reporting to ensure reliable and generalisable findings are required. In summary, the proposed benefits of hot yoga and areas of unknown/future research are presented in Fig. 2.

### Case Studies Associated with Hot Yoga

Although most hot yoga research details experimental investigations of the acute responses and chronic adaptations, there are several case studies which outline the negative consequences attributed to PA in hot conditions. These episodes of ill-health include cardiovascular dysfunction (e.g., sudden cardiac arrest [49] and acute myocardial infarction [50]) experienced during hot yoga. Hyponatremia has also been reported following hot yoga and the likely uneducated/misinformed consumption of 3.5 L of water [52]. Dermatological issues (e.g., photo urticaria) have been reported, likely due to the repetitive exposure to red light during modified hot yoga [54], as well as an account of psychosis (e.g., auditory and visual hallucinations, and paranoia) during a Bikram yoga instructors’ training program [51]. Whilst these studies provide an insight into the negative aspects associated with PA in heat stress, it is acknowledged there are few published reports and direct causes linked to the exercise/conditions are in some cases speculative. However, as with all forms of PA, many more hot yoga users likely experience minor incidences/illnesses which do not require hospitalisation/treatment (e.g., musculoskeletal issues, fatigue, headache, dizziness). Likewise, there are many contributing factors to these unfortunate circumstances, for example, underlying cardiac health problems and/or history of psychological issues, and thus future research should comprehensively document participant characteristics. Some illnesses outlined within these case studies may have been preventable with education and thus we provide evidence-based health and safety recommendations below to support safe and effective teaching and practice of hot yoga.

### Evidence-Based Health and Safety Recommendations

Despite the proposed benefits of hot yoga, additional heat stress during exercise has the potential to pose a risk to health, such as HRI. Those inexperienced or unaware of HRI symptoms, or who have pre-existing health conditions (e.g., cardiometabolic disease), likely face the greatest health risks and should seek general practitioner clearance prior to undertaking this form of exercise. Despite limited supporting research, survey data demonstrate adverse effects from practicing hot yoga, with symptoms including dizziness (60%), light-headedness (61%), nausea (35%) and dehydration (34%) in 157 participants [68]. Each of these symptoms may be precursors to exertional heat illnesses (EHI). The authors therefore advocate training and educating both participants/students and instructors/teachers of hot yoga on the prevention, symptom recognition and treatment of HRI and associated illnesses (e.g., EHI, EAH). Instructors/teachers have a duty of care to monitor the health of their participants during hot yoga and are well positioned to raise awareness of HRI and preventative strategies, such as encouraging adequate hydration practices prior to and following hot yoga [69]. We provide the following recommendations for safe hot yoga practice:

- As with all PA in hot and humid conditions, commencing yoga hydrated is recommended. Prior to commencing hot yoga, teachers and students are guided to drink approximately 5 mL per kg BM (i.e., 300–400 mL [70]) to ensure they are adequately hydrated. Individuals should familiarise themselves with their tolerance of pre-exercise fluid intake to ensure gastric comfort once exercise has commenced. Teachers and venues can support this practice with verbal reminders prior to classes and placing signs at the entrance to the venue.
- Following hot yoga, teachers and students should rehydrate, which may include electrolytes, to replenish those lost through sweating [71]. It is unnecessary to drink large volumes of fluid (i.e., > 3 L) during or immediately following hot yoga, given the sweat rates typically observed (1.5 ± 0.7 L), and cases of hyponatremia following excessive rehydration have been reported [52, 72]. Matching fluid intake to sweat loss, or up to 1.5 times sweat loss, is recommended in the hours following the class.
- Hydration behaviours before and during hot yoga are related to instructional encouragement [69]. Instructors therefore have a role in optimising hydration practices and helping to minimise the risk of adverse heat-related outcomes [69]. Instructors should be aware of the guidelines and monitor participants, in particular new students, reminding them of recommended drinking behaviours.
- During hot yoga, participants experiencing high levels of thermal discomfort (e.g., they feel “*as hot as they can stand*”), may prefer to take ‘savasana’ - a relaxing posture in a supine position. This may enable the individual to remain in the heat until the class is finished, but with a lower physiological strain and associated discomfort. Remaining in the heat will enable students to passively adapt to the thermal strain they are experiencing and support improved heat tolerance in subsequent classes [73].
- The class should be terminated with immediate removal from the heat source if intolerance symptoms arise, including dizziness, headaches or nausea. If symptoms persist, immediate cooling should be initiated. The most effective form of cooling is cold water immersion, with the objective of maximising the skin surface area being cooled [74].
- As with heat training within athletic populations, novice practitioners should ensure a gradual build up over the first ~ 7–14 sessions of hot yoga, and increase their level of effort as they become adapted to the hot ambient conditions [64, 65]. Individuals who are obese and/or have low fitness, sweat gland dysfunction, viral illness or diarrhoea, are at greater risk of developing EHI [74] and should seek medical approval prior to undertaking a hot yoga class.
- Fluctuations in females’ hormones across the menstrual cycle (possibly due to higher initial T_CORE_ within the luteal phase [75]), perimenopause, menopause, and post-menopause may cause differences in heat tolerance and heat sensitivity and thus, females may need to modify the intensity of the class to support adherence [76].
- Scientific evidence regarding the risks and benefits of practicing hot yoga is lacking [77]. As such, pregnant women should seek general practitioner advice prior to undertaking hot yoga, ensuring they are aware of environmental limits for exercise and heat stress during pregnancy (e.g. ensuring T_CORE_ does not rise by 2 °C from resting [78]) and be mindful of the impacts of heat exposure on foetal development [79]. If pregnant women are medically cleared to participate in hot yoga, they should consider class modifications (e.g., lower intensity, shorter duration, location within class to cooler regions) to minimise the elevations in T_CORE_, especially in the first trimester [80].

Hot yoga research primarily utilises female participants, who represent > 75% of the reported population, as is found in non-heated yoga literature [11]. This is notable, as it contrasts with a wider trend within the field of sport and exercise science [7, 8] and thermal human biology literature [9, 10], which primarily utilise male participants. Hot yoga therefore presents an alternate heat-based exercise approach, supporting investigation of the physiological, psychological and functional responses of females to heat stress. We recommend researchers routinely monitor and report females’ menstrual status and any impact on study outcomes when conducting hot yoga research. It should also be made clear during which phase of the menstrual cycle testing occurred and where possible, conduct trials using the four-phase approach [81]. We direct readers to the working guidelines for standards of practice for research on females [81] and where possible adopt gold standard procedures [82].

### Future Research Considerations

Whilst studies have investigated the responses to and adaptations following hot yoga, some weak experimental designs have been identified (e.g. studies forgoing non-heated exercise control group comparisons, crossover designs and RCT design). The existing evidence lacks measurements that provide mechanistic insights and validated procedures. Not all studies reported full data sets (e.g., for outcome measures, participant characteristics or adherence/completion statistics of prescribed hot yoga session duration/frequency), and/or considered the effects of participant bias or experience levels. Hot/Bikram yoga has not been comprehensively evaluated against other established thermal or exercise interventions, and thus the combined benefit of the exercise and thermal stress cannot be quantified against these elements independently. Related to this, the mechanistic basis for the thermal superimposition of the intervention is not well evidenced. At the current time experimental studies have not fully characterised the influence of exercise intensity or situational factors, such as the control of ambient conditions, size of the room and number of participants undertaking hot yoga at once, on the efficacy of the intervention. Each of these factors can influence an individual’s heat exchange with the environment and thus alter the physiological and/or psychological responses [55]. Future research investigating hot yoga should address these highlighted limitations and include current and former [2] emphasised recommendations within future research designs.

## Conclusion

Acute hot yoga increases core temperature and heart rate, but not energetic demands compared to other forms of non-heated yoga. Repeated (chronic) hot yoga practice appears to elicit some cardiometabolic (e.g., body composition, lipid profiles and macrovascular function) and functional adaptations applicable for health (e.g., bone mineral density, balance and flexibility) as well as potential physical performance improvements (e.g., submaximal exercise thresholds), although further confirmatory research is required. These effects occur without apparently impacting kidney function or sleep quality across healthy, sedentary and elite athlete populations. Hot yoga also presents promising, albeit inconclusive findings concerning its utility in alleviating psychological and affective disorders, and optimising markers of cognitive function. However, caution is advised following cases of ill-health and reports of heat illness. Accordingly, we provide evidence-based health and safety recommendations for safe and effective teaching and practice. Finally, hot yoga offers a large representation of female participants (76%), which provides a novel area for thermal physiology research to explore. Researchers should consider the highlighted limitations/recommendations to address experimental issues within future research designs.

## Data Availability

All data generated and analysed within this study are included in this published article and/or respective articles included in the systematic review.

## Abbreviations

A.U: Arbitrary unit
ADHD: Attention deficit hyperactivity disorder
BMD: Bone mineral density
TCORE: Core temperature
EE: Energy expenditure
EAH: Exercise-associated hyponatremia
EHI: Exertional heat illnesses
FMD: Flow mediated dilatation
HSP: Heat shock protein
HRI: Heat-related illness
HR: Heart rate
HDL: High-density lipoprotein
HIIT: High-intensity interval training
HOMA-IR: Homeostatic model assessment for insulin resistance
LDL: Low-density lipoprotein
V̇O2max: Maximal oxygen uptake
MeSH: Medical subject headings
METs: Metabolic equivalents
MVC: Maximal voluntary contraction
V̇O2: Oxygen uptake
PA: Physical activity
PICO: Population, Intervention, Comparison, and Outcome
PRISMA: Preferred Reporting Items for Systematic reviews and Meta-Analyses
PV: Plasma volume
PWV: Peak wave velocity
RCT: Randomised controlled trials
ROM: Range of motion
RPE: Rating of perceived exertion
RH: Relative humidity
RER: Respiratory exchange ratio
SD: Standard deviation
TTE: Time to exhaustion

## References

[1] Ross A, Thomas S. The health benefits of yoga and exercise: a review of comparison studies. J Altern Complement Med. 2010;16(1):3–12. 20105062 10.1089/acm.2009.0044

[2] Hewett ZL, Cheema BS, Pumpa KL, Smith CA. The effects of Bikram yoga on health: critical review and clinical trial recommendations. Evid Based Complement Alternat Med; 2015. p. 428427. 10.1155/2015/428427 PMC4609431 26504475

[3] Liguori G, American College of Sports Medicine. ACSM’s guidelines for exercise testing and prescription. Lippincott Williams & Wilkins; 2020.

[4] Pate JL, Buono MJ. The physiological responses to Bikram yoga in novice and experienced practitioners. Altern Ther Health Med. 2014;20(4):12–8. 25141359

[5] Bull FC, Al-Ansari SS, Biddle S, Borodulin K, Buman MP, Cardon G, Willumsen JF. World health organization 2020 guidelines on physical activity and sedentary behaviour. Br J Sports Med. 2020;54(24):1451–62. 33239350 10.1136/bjsports-2020-102955 PMC7719906

[6] Quandt E, et al. Heart rate and core temperature responses to Bikram yoga. Gundersen Med J. 2015;8:3.

[7] Costello JT, Bieuzen F, Bleakley CM. Where are all the female participants in sports and exercise medicine research? Eur J Sport Sci. 2014;14(8):847–51. 24766579 10.1080/17461391.2014.911354

[8] Cowley ES, Olenick AA, McNulty KL, Ross EZ. Invisible sportswomen: the sex data gap in sport and exercise science research. Women Sport Phys Activity J. 2021;29(2):146–51.

[9] Hutchins KP, Borg DN, Bach AJE, Bon JJ, Minett GM, Stewart IB. Female (under) representation in exercise thermoregulation research. Sports Med. 2021, 7. 10.1186/s40798-021-00334-6 PMC8219822 34156570

[10] Kelly MK, Smith ES, Brown HA, Jardine WT, Convit L, Bowe SJ, Condo D, Guy JH, Burke LM, Periard JD, Snip RMJ, Snow RJ, Carr AJ. Auditing the representation of female versus males in heat adaptation research. Int J Sport Nutr Exerc Metab. 2024;34(2):111–21. 38211577 10.1123/ijsnem.2023-0186

[11] Park CL, Braun T, Siegel T. Who practices yoga? A systematic review of demographic, health-related, and psychosocial factors associated with yoga practice. J Behav Med. 2015;38:460–71. 25627668 10.1007/s10865-015-9618-5

[12] Page MJ, McKenzie JE, Bossuyt PM, Boutron I, Hoffmann TC, Mulrow CD, Moher D. The PRISMA 2020 statement: an updated guideline for reporting systematic reviews. bmj. 2021, 372. 10.1136/bmj.n71 PMC8005924 33782057

[13] Methley AM, Campbell S, Chew-Graham C, McNally R, Cheraghi-Sohi S, PICO. PICOS and SPIDER: a comparison study of specificity and sensitivity in three search tools for qualitative systematic reviews. BMC Health Serv Res. 2014;14:579. 10.1186/s12913-014-0579-0. 25413154 10.1186/s12913-014-0579-0 PMC4310146

[14] Alrefai H, Mathis SL, Hicks SM, Pivovarova AI, MacGregor GG. Salt and water balance after sweat loss: A study of Bikram yoga. Physiological Rep. 2020;8(22):e14647. 10.14814/phy2.14647 PMC7683807 33230967

[15] Boyd CN, Lannan SM, Zuhl MN, Mora-Rodriguez R, Nelson RK. Objective and subjective measures of exercise intensity during thermo-neutral and hot yoga. Appl Physiol Nutr Metab. 2018;43(4):397–402. 29169011 10.1139/apnm-2017-0495

[16] Dysart A, Harden SM. Effects of temperature and tempo: evaluating how much time in a typical community-based yoga class is moderate-intensity aerobic activity. Int J Environ Res Public Health. 2023;20(3):2349. 36767717 10.3390/ijerph20032349 PMC9915918

[17] Fritz ML, Grossman AM, Mukherjee A, Hunter SD, Tracy BL. Acute metabolic, cardiovascular, and thermal responses to A single session of Bikram yoga: 593 board# 8 May 28, 3: 30 PM-5: 00 PM. Medicine & Science in Sports & Exercise. 2014;46(5S):146–7.

[18] Hurtado M, Valladares C, Eblen-Zajjur A, Rodriguez-Fernandez M. Acute cardiovascular responses to a session of Bikram yoga: a pilot uncontrolled trial. J Altern Complement Med. 2019;25(4):398–405. 30698456 10.1089/acm.2018.0261

[19] Lambert BS, Miller KE, Delgado DA, Chaliki K, Lee J, Bauza G, McCulloch PC. Acute physiologic effects of performing yoga in the heat on energy expenditure, range of motion, and inflammatory biomarkers. Int J Exerc Sci. 2020;13(3):802. 32509120 10.70252/AKMZ9424 PMC7241641

[20] Mathis SL, Pivovarova AI, Hicks SM, Alrefai H, MacGregor GG. Calcium loss in sweat does not stimulate PTH release: A study of Bikram hot yoga. Complement Ther Med. 2020;51:102417. 32507433 10.1016/j.ctim.2020.102417

[21] Szabo A, Nikházy L, Tihanyi B, Boros S. An in-situ investigation of the acute effects of Bikram yoga on positive-and negative affect, and state-anxiety in context of perceived stress. J Mental Health. 2017;26(2):156–60. 10.1080/09638237.2016.1222059 27809649

[22] Abel AN, Lloyd LK, Williams JS, Miller BK. Physiological characteristics of Long-Term Bikram yoga practitioners. J Exerc Physiol Online. 2012;15(5):32–9.

[23] Bourbeau KC, Moriarty TA, Bellovary BN, Bellissimo GF, Ducharme JB, Haeny TJ, Zuhl MN. Cardiovascular, cellular, and neural adaptations to hot yoga versus normal-temperature yoga. Int J Yoga. 2021;14(2):115–26. 34188383 10.4103/ijoy.IJOY_134_20 PMC8191229

[24] Bordman R, Meaney C, Telner D. The effects of hot yoga on kidney function: an observational pilot and feasibility study. Int J Yoga Therapy. 2022;32(2022):Article–1. 10.17761/2022-D-21-00001 35100415

[25] Flehr, A., Barton, C., Coles, J., Gibson, S. J., Lambert, G. W., Lambert, E. A., …Dixon, J. B. MindinBody-feasibility of vigorous exercise (Bikram yoga versus high intensity interval training) to improve persistent pain in women with a history of trauma: a pilot randomized control trial. BMC complementary and alternative medicine. 2019;19:1–16. 10.1186/s12906-019-2642-1 PMC6714085 31464643

[26] Fritz K, O’Connor PJ. Effects of a 6 week yoga intervention on executive functioning in women screening positive for adult ADHD: A pilot study. Front Sports Act Living. 2022;4:746409. 35280225 10.3389/fspor.2022.746409 PMC8908201

[27] Hart CEF, Tracy BL. Yoga as steadiness training: effects on motor variability in young adults. J Strength Conditioning Res. 2008;22(5):1659–69. 10.1519/JSC.0b013e31818200dd 18714217

[28] Hewett ZL, Ransdell LB, Gao Y, Petlichkoff LM, Lucas S. An examination of the effectiveness of an 8-week Bikram yoga program on mindfulness, perceived stress, and physical fitness. J Exerc Sci Fit. 2011;9(2):87–92.

[29] Hewett ZL, Pumpa KL, Smith CA, Fahey PP, Cheema BS. Effect of a 16-week Bikram yoga program on perceived stress, self-efficacy and health-related quality of life in stressed and sedentary adults: A randomised controlled trial. J Sci Med Sport. 2018;21(4):352–7. 28866110 10.1016/j.jsams.2017.08.006

[30] Hewett ZL, Pumpa KL, Smith CA, Fahey PP, Cheema BS. Effect of a 16-week Bikram yoga program on heart rate variability and associated cardiovascular disease risk factors in stressed and sedentary adults: A randomized controlled trial. BMC Complement Altern Med. 2017;17:1–11. 28431533 10.1186/s12906-017-1740-1 PMC5399826

[31] Hewett ZL, Pumpa KL, Smith CA, Fahey PP, Cheema BS. Predictors of and barriers to adherence in a 16-week randomised controlled trial of Bikram yoga in stressed and sedentary adults. Complement Ther Med. 2019;42:374–80. 10.1016/j.ctim.2018.12.015. 30670270 10.1016/j.ctim.2018.12.015

[32] Hopkins LB, Medina JL, Baird SO, Rosenfield D, Powers MB, Smits JAJ. Heated Hatha yoga to target cortisol reactivity to stress and affective eating in women at risk for Obesity-Related illnesses: A randomized controlled trial. J Consult Clin Psychol. 2016;84(6):558–64. 26963599 10.1037/ccp0000091 PMC4873332

[33] Hui BPH, Parma L, Kogan A, Vuillier L. Hot yoga leads to greater Well-being: A Six-week Experience-sampling RCT in healthy adults. Psychosocial Intervention. 2022;31(2):67–82. 37360056 10.5093/pi2022a4 PMC10268545

[34] Hunter SD, Dhindsa MS, Cunningham E, Tarumi T, Alkatan M, Tanaka H. Improvements in glucose tolerance with Bikram yoga in older obese adults: a pilot study. J Bodyw Mov Ther. 2013;17(4):404–7. 24138995 10.1016/j.jbmt.2013.01.002

[35] Hunter SD, Dhindsa MS, Cunningham E, Tarumi T, Alkatan M, Nualnim N, Tanaka H. The effect of Bikram yoga on arterial stiffness in young and older adults. J Altern Complement Med. 2013;19(12):930–4. 23738677 10.1089/acm.2012.0709

[36] Hunter SD, Dhindsa MS, Cunningham E, Tarumi T, Alkatan M, Nualnim N, Tanaka H. Impact of hot yoga on arterial stiffness and quality of life in overweight/obese adults. J Phys Activity Health. 2016;13(12):1360–3. 10.1123/jpah.2016-0170 27633625

[37] Hunter, S. D., Dhindsa, M. S., Cunningham, E., Tarumi, T., Alkatan, M., Nualnim, N., … Tanaka, H. The effect of Bikram yoga on endothelial function in young and middle-aged and older adults. Journal of bodywork and movement therapies. 2017;21(1):30–34. 10.1016/j.jbmt.2016.06.004 28167186

[38] Hunter SD, Laosiripisan J, Elmenshawy A, Tanaka H. Effects of yoga interventions practised in heated and thermoneutral conditions on endothelium-dependent vasodilatation: the Bikram yoga heart study. Exp Physiol. 2018;103(3):391–6. 29349832 10.1113/EP086725

[39] Hunter SD, Laosiripisan J, Elmenshawy A. Effects of heated and thermoneutral yoga interventions on arterial stiffness in middle-aged adults. Complement Ther Med. 2018;40:113–5. 30219434 10.1016/j.ctim.2018.08.005

[40] Hunter SD, Kavouras SA, Rahimi M. Exploring heated exercise as a means of preventing the deleterious effects of high-sodium intake in black women. Am J Physiol Heart Circ Physiol. 2023;324(6):H833–9. 37027326 10.1152/ajpheart.00699.2022

[41] Kudesia RS, Bianchi MT. Decreased nocturnal awakenings in young adults performing Bikram yoga: A Low-Constraint home sleep monitoring study. International Scholarly Research Network Neurology; 2012. p. 153745. 10.5402/2012/153745 PMC3345216 22577578

[42] La Rocque CL, Mazurka R, Stuckless TJ, Pyke K, Harkness KL. Randomized controlled trial of Bikram yoga and aerobic exercise for depression in women: efficacy and stress-based mechanisms. J Affect Disord. 2021;280:457–66. 33242717 10.1016/j.jad.2020.10.067

[43] Medina J, Hopkins L, Powers M, Baird SO, Smits J. The effects of a Hatha yoga intervention on facets of distress tolerance. Cogn Behav Ther. 2015;44(4):288–300. 25952547 10.1080/16506073.2015.1028433 PMC4681579

[44] Nyer, M., Hopkins, L. B., Farabaugh, A., Nauphal, M., Parkin, S., McKee, M. M., …Mischoulon, D. Community-delivered heated hatha yoga as a treatment for depressive symptoms: an uncontrolled pilot study. The Journal of Alternative and Complementary Medicine. 2019;25(8):814–823. 10.1089/acm.2018.0365 PMC6763961 31290694

[45] Nyer, M. B., Hopkins, L. B., Nagaswami, M., Norton, R., Streeter, C. C., Hoeppner,B. B., … Mischoulon, D. A Randomized Controlled Trial of Community-Delivered Heated Hatha Yoga for Moderate-to-Severe Depression. The Journal of clinical psychiatry. 2023;84(6):49607. 10.4088/JCP.22m14621 PMC12160574 37883245

[46] Perrotta AS, White MD, Koehle MS, Taunton JE, Warburton DER. Efficacy of hot yoga as a heat stress technique for enhancing plasma volume and cardiovascular performance in elite female field hockey players. J Strength Conditioning Res. 2018;32(10):2878–87. 10.1519/JSC.0000000000002705 29979281

[47] Sangiorgio SN, Mukherjee AK, Lau NW, Mukherjee A, Mukhopadhyay P, Ebramzadeh E. Optimization of physical activity as a countermeasure of bone loss: a 5-year study of Bikram yoga practice in females. Health. 2014;6(11):1124–32.

[48] Tracy BL, Hart CEF. Bikram yoga training and physical fitness in healthy young adults. J Strength Conditioning Res. 2013;27(3):822–30. 10.1519/JSC.0b013e31825c340f 22592178

[49] Boddu P, Patel S, Shahrrava A. Sudden cardiac arrest from heat stroke: hidden dangers of hot yoga. Am J Med. 2016;129(8):e129–30. 27107927 10.1016/j.amjmed.2016.03.030

[50] Ferrera C, Echavarría-Pinto M, Nuñez-Gil I, Alfonso F. Bikram yoga and acute myocardial infarction. J Am Coll Cardiol. 2014;63(12):1223. 24509268 10.1016/j.jacc.2013.10.091

[51] Lu JS, Pierre JM. Psychotic episode associated with Bikram yoga. Am J Psychiatry. 2007;164(11):1761–1761. 17974947 10.1176/appi.ajp.2007.07060960

[52] Reynolds CJ, Cleaver BJ, Finlay SE. Exercise associated hyponatraemia leading to tonic-clonic seizure. BMJ Case Reports. 2012;2012:bcr0820114625. 10.1136/bcr.08.2012.4625 PMC3433526 22927272

[53] Sakurai, H., Norton, R. J., Fisher, L. B., Nagaswami, M. V., Streeter, C. C., Meyer,A. K., … Nyer, M. B. A Patient With Electroconvulsive Therapy–resistant Major Depressive Disorder With a Full Response to Heated Yoga: A Case Report. Journal of Psychiatric Practice^®^. 2021;27(6):486–491. 10.1097/PRA.0000000000000587 34768274

[54] Takeuchi A, Fukumoto T, Nishigori C. Photo urticaria caused by exposure to LED emitting 633-nm wavelength during hot yoga exercise. PhotoDermatol PhotoImmunol PhotoMed. 2022;38(4):395–6. 34882840 10.1111/phpp.12757

[55] Jay O. Unravelling the true influences of fitness and sex on sweating during exercise. Exp Physiol. 2014;99(10):1265–6. 25274337 10.1113/expphysiol.2014.080994

[56] James CA, Hayes M, Willmott AG, Gibson OR, Flouris AD, Schlader ZJ, Maxwell NS. Defining the determinants of endurance running performance in the heat. Temperature. 2017;4(3):314–29. 10.1080/23328940.2017.1333189 PMC5605161 28944273

[57] Aaron EA, Seow KC, Johnson BD, Dempsey JA. Oxygen cost of exercise hyperpnea: implications for performance. J Appl Physiol. 1992;72(5):1818–25. 1601791 10.1152/jappl.1992.72.5.1818

[58] Racinais S, Oksa J. Temperature and neuromuscular function. Scand J Med Sci Sports. 2010;20(3):1–18. 21029186 10.1111/j.1600-0838.2010.01204.x

[59] Mornas A, Racinais S, Brocherie F, Alhammoud M, Hager R, Desmedt Y, Guilhem G. Faster early rate of force development in warmer muscle: an in vivo exploration of fascicle dynamics and muscle-tendon mechanical properties. Am J Physiology-Regulatory Integr Comp Physiol. 2022;323(1):R123–32. 10.1152/ajpregu.00280.2021 35579335

[60] Szabo A, Gáspár Z, Kiss N, Radványi A, Gaskins RB, Jennings E, Thind H et al. Acute and cumulative effects of Vinyasa yoga on affect and stress among college students participating in an eight-week yoga program: A pilot study. Int J Yoga Ther. 2014;24:63–70. 25858652

[61] Gaskins R, Jennings E, Thind H, Becker B, Bock B. Acute and cumulative effects of Vinyasa yoga on affect and stress among college students participating in an eight-week yoga program: A pilot study. Int J Yoga Therapy. 2014;24(1):63–70. 25858652

[62] Reed J. Self-reported morningness-eveningness related to positive affect-change associated with a single session of Hatha yoga. Int J Yoga Ther. 2014;24:79–85. 25858654

[63] Mandsager K, Harb S, Cremer P, Phelan D, Nissen SE, Jaber W. Association of cardiorespiratory fitness with long-term mortality among adults undergoing exercise treadmill testing. JAMA Netw Open. 2018;1(6):e183605–183605. 30646252 10.1001/jamanetworkopen.2018.3605 PMC6324439

[64] Racinais S, Casa D, Brocherie F, Ihsan M. Translating science into practice: the perspective of the Doha 2019 IAAF world championships in the heat. Front Sports Act Living. 2019;1:39. 33344962 10.3389/fspor.2019.00039 PMC7739640

[65] Gibson OR, James CA, Mee JA, Willmott AG, Turner G, Hayes M, Maxwell NS. Heat alleviation strategies for athletic performance: a review and practitioner guidelines. Temperature. 2020;7(1):3–36. 10.1080/23328940.2019.1666624 PMC7053966 32166103

[66] Kim K, Monroe JC, Gavin TP, Roseguini BT. Skeletal muscle adaptations to heat therapy. J Appl Physiol. 2020;128(6):1635–42. 32352340 10.1152/japplphysiol.00061.2020 PMC7311689

[67] Brunt VE, Minson CT. Heat therapy: mechanistic underpinnings and applications to cardiovascular health. Journal of Applied Physiology; 2021. 10.1152/japplphysiol.00141.2020 PMC8285605 33792402

[68] Mace C, Eggleston B. Self-reported benefits and adverse outcomes of hot yoga participation. Int J Yoga Therapy. 2016;26(1):49–53. 10.17761/1531-2054-26.1.49 27797657

[69] Firebaugh CJM, Eggleston B. Hydration and hot yoga: encouragement, behaviors, and outcomes. Int J Yoga. 2017;10(2):107–9. 28546683 10.4103/ijoy.IJOY_8_17 PMC5433110

[70] Burke LM. Nutritional needs for exercise in the heat. Comp Biochem Physiol A: Mol Integr Physiol. 2001;128(4):735–48. 11282317 10.1016/s1095-6433(01)00279-3

[71] American College of Sports Medicine. ACSM’s guidelines for exercise testing and prescription. 8th ed. New York, NY: Lippincott Williams & Wilkins; 2009.

[72] Bailowitz Z, Grams R 2, Teeple D, Hew-Butler T. Exercise-associated hyponatremia in a lactating female. Clin J Sport Med. 2017;27(4):e55–7. 10.1097/JSM.0000000000000344 28653967

[73] Racinais S, Wilson MG, Gaoua N, Périard JD. Heat acclimation has a protective effect on the central but not peripheral nervous system. J Appl Physiol. 2017;123(4):816–24. 28684590 10.1152/japplphysiol.00430.2017

[74] Armstrong LE, Casa DJ, Millard-Stafford M, Moran DS, Pyne SW, Roberts WO. Exertional heat illness during training and competition. Medicine & Science in Sports & Exercise. 2007;39(3):556–72. 10.1249/MSS.0b013e31802fa199 17473783

[75] Giersch GE, Morrissey MC, Katch RK, Colburn AT, Sims ST, Stachenfeld NS, Casa DJ. Menstrual cycle and thermoregulation during exercise in the heat: A systematic review and meta-analysis. J Sci Med Sport. 2020;23(12):1134–40. 32499153 10.1016/j.jsams.2020.05.014

[76] Marsh SA, Jenkins DG. Physiological responses to the menstrual cycle: implications for the development of heat illness in female athletes. Sports Med. 2002;32:601–14. 12141881 10.2165/00007256-200232100-00001

[77] Mace C, Eggleston B. Hot yoga and pregnancy: Establishing participation rates and areas for further investigation. Int J Health Wellness Soc. 2019;9(2):39.

[78] Ravanelli N, Casasola W, English T, Edwards KM, Jay O. Heat stress and fetal risk. Environmental limits for exercise and passive heat stress during pregnancy: a systematic review with best evidence synthesis. Br J Sports Med. 2019;53(13):799–805. 29496695 10.1136/bjsports-2017-097914

[79] Edwards MJ, Saunders RD, Shiota K. Effects of heat on embryos and foetuses. Int J Hyperth. 2003;19(3):295–324. 10.1080/0265673021000039628 12745973

[80] Moretti ME, Bar-Oz B, Fried S, Koren G. Maternal hyperthermia and the risk for neural tube defects in offspring: systematic review and meta-analysis. Epidemiology. 2005;16(2):216–9. 15703536 10.1097/01.ede.0000152903.55579.15

[81] Elliott-Sale, K. J., Minahan, C. L., de Jonge, X. A. J., Ackerman, K. E., Sipilä,S., Constantini, N. W., … Hackney, A. C. Methodological considerations for studies in sport and exercise science with women as participants: a working guide for standards of practice for research on women. Sports Medicine. 2021;51(5):843–861. 10.1007/s40279-021-01435-8 PMC8053180 33725341

[82] Smith ES, McKay AK, Ackerman KE, Harris R, Elliott-Sale KJ, Stellingwerff T, Burke LM. Methodology review: a protocol to audit the representation of female athletes in sports science and sports medicine research. Int J Sport Nutr Exerc Metab. 2022;32(2):114–27. 35168200 10.1123/ijsnem.2021-0257

